# Optical Properties of GaSb Nanofibers

**DOI:** 10.1007/s11671-010-9739-2

**Published:** 2010-08-21

**Authors:** Xiuli Zhou, Wei Guo, Alejandro G Perez-Bergquist, Qiangmin Wei, Yanbin Chen, Kai Sun, Lumin Wang

**Affiliations:** 1 School of Physical Electronics, University of Electronic Science and Technology of China, 610054 Chengdu, China; 2 Department of Materials Science and Engineering, University of Michigan, 48109 Ann Arbor, MI, USA; 3 Department of Electrical Engineering and Computer Science, University of Michigan, 48109 Ann Arbor, MI, USA; 4 Department of Nuclear Engineering and Radiological Sciences, University of Michigan, 48109 Ann Arbor, MI, USA

**Keywords:** GaSb, Ion beam irradiation, Raman scattering, Photoluminescence (PL)

## Abstract

Amorphous GaSb nanofibers were obtained by ion beam irradiation of bulk GaSb single-crystal wafers, resulting in fibers with diameters of ~20 nm. The Raman spectra and photoluminescence (PL) of the ion irradiation-induced nanofibers before and after annealing were studied. Results show that the Raman intensity of the GaSb LO phonon mode decreased after ion beam irradiation as a result of the formation of the amorphous nanofibers. A new mode is observed at ~155 cm^-1^ both from the unannealed and annealed GaSb nanofiber samples related to the A_1g_ mode of Sb–Sb bond vibration. Room temperature PL measurements of the annealed nanofibers present a wide feature band at ~1.4–1.6 eV. The room temperature PL properties of the irradiated samples presents a large blue shift compared to bulk GaSb. Annealed nanofibers and annealed nanofibers with Au nanodots present two different PL peaks (400 and 540 nm), both of which may originate from Ga or O vacancies in GaO. The enhanced PL and new band characteristics in nanostructured GaSb suggest that the nanostructured fibers may have unique applications in optoelectronic devices.

## Introduction

III–V semiconductors are increasingly important for electronic and optoelectronic devices due to their high electron mobility and direct bandgap. And nanostructured semiconductors have been attracting widespread attention for their unique quantum-confined nanoscale properties. In particular, the optical properties of nanoscale semiconductors are seen as a key to the future of optoelectronic device fabrication [1,2]. One material that has received substantial attention in this field is gallium antimonide (GaSb), a very attractive material system for lasers, modulators and detectors because the fundamental gap of GaSb lies close to the 1.55 μm low attenuation window of silica optical fibers. GaSb is also an ideal substrate for some longer wavelength lasers and photodetectors [3-5], low-power-consumption electronic devices [6], optoelectronic devices with varying wavelengths [7] and ordered semiconductor quantum dots [8]. For these reasons, it is necessary to continue to improve our understanding of GaSb and to get deep understanding of its physical properties.

Some studies using ion accelerators [9], low-energy-focused Ga^+^ ion beams (FIB) [10-12] and high-energy Au^+^ and Kr^+^ ion beams [13] have shown that ion irradiation of GaSb under appropriate implantation conditions results in the formation of porous surface structures. To date, however, there has been little investigation on the optical characteristics of these porous materials after ion bombardment and annealing [14,15]. In this communication, we present the formation of nanofibers on the surface of GaSb single crystals by low-energy-focused Ga^+^ and high-energy Au^+^ and Kr^+^ ion beam irradiation. And thermal annealing was conducted to crystallize the GaSb nanofibers. We analyze the optical properties of the GaSb nanofiber semiconductors by means of Raman scattering and photoluminescence (PL). It shows that the substrate signal of the GaSb LO mode appears at 237 cm^-1^, and the nanostructured GaSb samples show peaks around ~155 cm^-1^, which can be assigned to the A_1g_ peak of crystalline Sb. The visible room temperature PL spectrum of the annealed nanofibers demonstrates an increase in luminescent intensity, and the low temperature (15 K) PL spectrum presents two new PL peaks (400 and 540 nm) when compared to bulk GaSb.

## Experimental

GaSb single-crystal wafers with (100) orientation were irradiated with a 30 keV focused Ga^+^ ion beam at room temperature. The evolution of the surface morphology of GaSb was monitored in situ in an FEI Nova 200 Nanolab FIB/SEM dual beam system. Conventional broad ion beam irradiation of GaSb using 150 keV Kr^+^ ions (with a beam diameter of ~25 mm) and 1 MeV Au^+^ ions was also conducted.

For the annealing study, parts of the irradiated samples were annealed at 250 and 350°C for 10 min in a conventional open tube furnace. Irradiated samples, as well as irradiated samples coated with Au thin film, were annealed at 600°C for 10 min.

Raman scattering experiments were demonstrated in backscattering geometry from the (100) sample surface at room temperature using a 633 nm He–Ne laser as an excitation source coupled to the commercial Raman spectrometry system. For III–V compound semiconductors of the zinc-blende crystal structure, Raman spectra generally show two peaks. The lower-frequency peak corresponds to TO phonons, and the higher frequency peak corresponds to LO phonons. In backscattering, only LO phonons appear in the (100) direction [16]. The laser output power was fixed at 200 mW so as to avoid excess heating of the samples. The scattered light was analyzed using a standard double-grating spectrometer in the photon-counting mode, whose spectral resolution is better than 2 cm^-1^. Room temperature PL characteristics of the GaSb nanofibers and bulk GaSb were also obtained using the 633 nm He–Ne laser. Low temperature PL characteristics of the bulk GaSb, annealed GaSb nano fibers and GaSb nano fibers coated with Au were conducted using a 325-nm He–Cd laser with output power 50 mW.

## Results and Discussion

**Figure 1:**
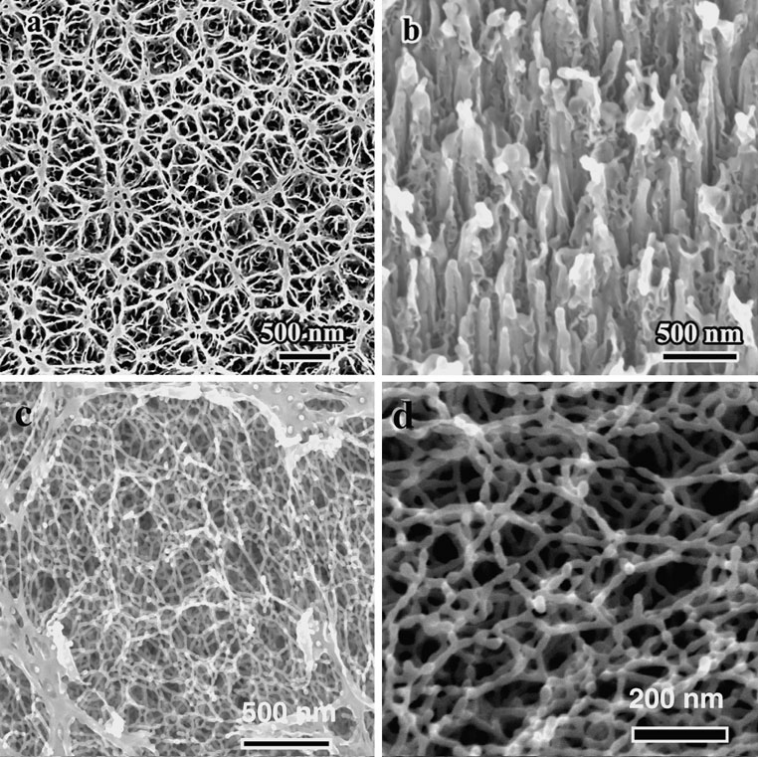
**SEM images of GaSb nanofibers**. **a** Under normal Ga^+^ ion beam bombardment at 30 keV. **b** With an incident angle of 70°, Ga^+^ ion beam bombardment at 30 keV. **c** and **d** Under normal Kr^+^ ion beam bombardment at 150 keV.

**Figure 2:**
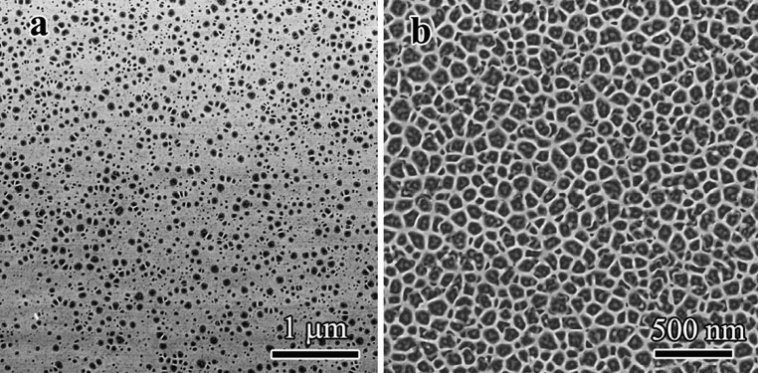
**SEM images of GaSb under different ion beam irradiation fluences**. **a** GaSb irradiated with Ga^+^ ions to a fluence of 5.2 × 10^15^ cm^-2^ under normal bombardment. Only individual voids form at low dose. **b** GaSb irradiated with Ga^+^ ions to a fluence of 1.1 × 10^16^ cm^-2^ under normal bombardment. Fiber networks form at higher doses.

Figure 1a, b present in situ scanning electronic microscope (SEM) images showing the morphology of GaSb under 30 keV Ga^+^ ion beam bombardment with incident angles of 0° and 70°, respectively. Figure 1c, d present SEM images showing the morphology of GaSb with Kr^+^ irradiation at 150 keV. It was found that the surface quickly evolved into a high density network of uniformly spaced GaSb nanofibers. Parts of the nanofibers were connected together to form a flake-like structure. The diameters of the relatively uniform nanofibers measured by the SEM is ~20 nm (Figure 1a, b). Increasing the irradiation time, which corresponds to an increase in irradiation fluence, decreases the size of the nanofibers. At low fluences, only small voids are formed, as shown in Figure 2a. With continuous bombardment, these voids coalesce and subsequently form fiber-like networks, as shown in Figure 2b. The formation of the GaSb nanofibers can be attributed to the accumulation of atomic damage created by energetic ions [13], with redeposition, viscous flow, and curvature-dependent sputtering also contributing to the morphological evolution of the fibers [17-20].

**Figure 3:**
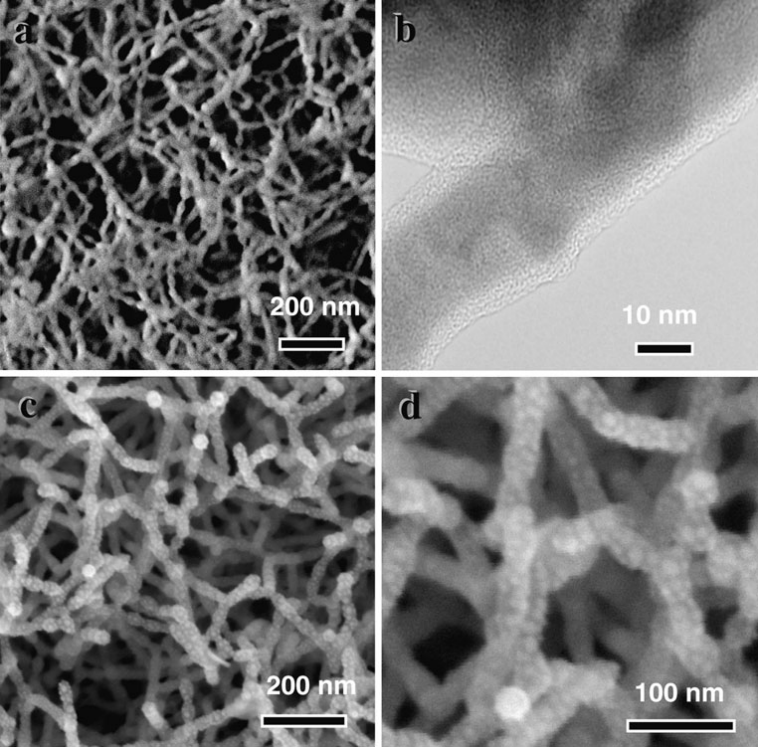
**SEM and TEM images of GaSb nanofibers formed with Au^+^ ions irradiation after annealed at 600°C for 10 min**. **a** SEM image of the annealed GaSb nanofibers **b** TEM image of the annealed GaSb nanofibers. **c**, **d** SEM images of Au-coated GaSb nanofibers after annealed at 600°C for 10 min.

Figure 3 shows SEM and TEM images of the GaSb nanofibers formed with Au^+^ ions irradiation after annealing at 600°C for 10 min. The annealed fibers exhibit a clear core–shell structure, as shown in Figure 3b. Since oxidation occurs during the annealing process, the composition of the shell layers is expected to be some form of gallium oxide. Figure 3c, d show the annealed, Au-coated GaSb fibers, which present Au nanodots distributed on the surface of the nanofibers.

**Figure 4:**
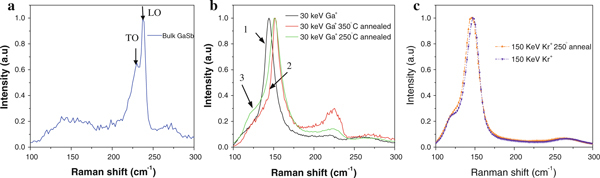
**a Raman spectrum of bulk GaSb**. **b** Raman spectra of GaSb nanofibers formed by 30 keV Ga^+^ ion irradiation. Included are spectra for: (*1*) unannealed nanofibers; (*2*) 350°C annealed nanofibers; (*3*) 250°C annealed nanofibers. **c** Raman spectra of GaSb nanofibers formed by 150 keV Kr^+^ ion irradiation.

Figure 4a shows the room temperature Raman spectrum of the bulk GaSb wafer. A strong peak was found at 237 cm^-1^ and a weak peak at 230 cm^-1^, which are the LO and TO modes, respectively. As mentioned above, only the LO mode is allowed for (100) oriented material, and the TO mode is forbidden [21]. However, a small peak due to the TO mode is also observed here, probably due to a slight substrate misorientation or imperfection. This phenomenon has also been observed by other semiconductors with (100) orientation [22].

The curves in Figure 4b present the Raman peaks of GaSb nanofibers irradiated by a 30 keV Ga^+^ ion beam. Raman spectroscopy was performed on both unannealed and annealed samples, at temperatures of 350 and 250°C for 10 min. From the spectra, we can see that the intensity of the LO modes becomes weaker after Ga^+^ irradiation. Figure 4b-1 shows the unannealed sample, where the LO mode red shifts to ~220 cm^-1^ and its intensity almost approaches zero. This means that the GaSb nanofibers become amorphous by Ga^+^ ion irradiation. Figure 4b-2 and 4b-3 present the spectra for the annealed samples, in which the LO mode red shifts to ~225 cm^-1^ and the full widths at half maximum (FWHM) were broadened in comparison with bulk GaSb spectrum. The stronger intensity of the LO modes means the amorphous nanofibers became crystalline through the annealing process. Such behavior of the Raman peak red shift and broadening can be explained by the phonon confinement effect [23]. Figure 4c presents the Raman peaks of GaSb nanofibers irradiated by 150 keV Kr^+^ ions, both unannealed and annealed at the temperature of 250°C. From the spectra, we cannot find the mode of GaSb from the two curves. These alterations of the LO mode by ion implantation on the crystalline structure are also attributed to the disordering of the crystalline structure. Because the decay of translational symmetry relaxes the momentum conservation, all photons in the Brillouin zone participate in ordered Raman scattering. These will generally induce the shift of the LO mode to a lower energy and cause asymmetric broadening [24].

At the same time, a new strong peak is observed at around 155 cm^-1^ for the samples after Ga^+^ and Kr^+^ ion beam bombardment as shown in Figure 4b, c. The intensity of the peaks is comparable to that of the LO modes of the bulk GaSb sample. This anomalous phenomenon is unique to GaSb. Kim et al. [15] conducted Rutherford back scattering (RBS) measurements on GaSb samples implanted with Ga^+^ ions and found that Sb atoms are deficient in the surface region of the irradiated areas. This phenomenon may be caused by the selective sputtering of Sb atoms during the ion bombardment process. However, recent work has shown that the thermal annealing of GaSb nanofibers results in a complete chemical decomposition of the nanofibers into crystalline Sb cores surrounded by amorphous GaO_x_ shells [25], which is consistent with our TEM data shown in Figure 3b. In the Sb crystal, there is a Raman peak at around 155 cm^-1^[13], which is related to the A_1g_ (150 cm^-1^) phonon of Sb. Carles et al. [26] have also observed Raman peaks at the same frequency on nonstoichiometric amorphous GaSb films and assigned this peak as the A_1g_ mode due to Sb–Sb bond vibration. Therefore, we can conclude that the Raman peak is related to Sb–Sb bond vibrations rather than other modes.

In order to study the characteristics of the amorphous and crystalline nanofibers, we compare the Raman spectra of Ga^+^ bombarded samples annealed at 250 and 350°C for 10 min and Kr^+^ bombarded samples annealed at 250°C, respectively. For the as-irradiated sample, as shown in Figure 4b-1, no distinct modes of GaSb were observed due to the amorphous state of the material. After annealing, the LO modes of GaSb are observed at around 225 cm^-1^, as shown in Figure 4b-2 and 4b-3. However, the LO mode from the sample annealed at 350°C was stronger than that from sample annealed at 250°C, showing that the LO mode of nanostructured GaSb increased with increasing annealing temperature, which means that the level of crystallinity of the nanofibers is still low after low temperature annealing. However, as shown in Figure 4c, there is no mode for GaSb. The networks of nanofibers induced by 150 keV Kr^+^ ion irradiation were more obvious on the GaSb surface, so the anomalous annealing behavior may be attributable to the thicker fiber layer forming underneath the material surface.

On the other hand, the FWHM of the 250°C annealed sample is wider than that of the 350°C annealed sample, with a peak at 155 cm^-1^ as shown in Figure 4b. This is again due to the formation of Sb crystal during annealing.

**Figure 5:**
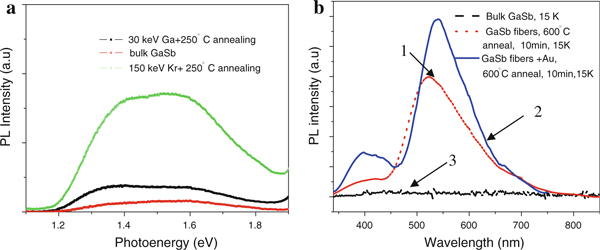
**a Room temperature PL intensity spectra for bulk GaSb and GaSb nanofibers annealed at 250°C**. **b** Low temperature (15 K) PL intensity for bulk GaSb, GaSb nanofibers annealed at 600°C and GaSb nanofibers coated with a thin Au film and then annealed at 600°C.

Figure 5a presents the room temperature PL characteristics of bulk GaSb and the annealed GaSb nanofibers. We can observe an enhancement of the PL in the range of 1.4–1.6 eV for the annealed 30 keV Ga^+^ and 150 keV Kr^+^ ion bombarded samples. Compared with bulk GaSb with a direct bandgap of ~0.72 eV at room temperature, the PL spectrum of the ion bombarded samples shows a blue shift in the bandgap. It seems likely that the PL mechanism of the GaSb nanofibers is similar to that of porous silicon [27]. Specifically, the exact treatment of this PL must be described quantum mechanically in terms of photons. For GaSb, the Bohr radius is about 20.46 nm [28], while nanofibers in GaSb are ~20 nm in diameter. According to the effective-mass approximation, there is an energetic blue shift ΔE that originates from nanoscale size effects of the GaSb nanofibers. On the other hand, ion irradiation-induced spatial separation of the bulk GaSb leads to an extremely sparse distribution of material compared with bulk GaSb. The networks of nanofibers are connected with air gaps in between, forming an inhomogeneous environment. When the fibers are irradiated by the laser, we can consider an emitting dipole in the nanofibers, and then the fields generated by the substrate include the dipole field E_0_ from pure GaSb and a scattered field E_s_ from the nanoscale, inhomogeneous GaSb fiber networks. As a result, there is an extra energy that results in the blue shift observed in our PL measurement. Figure 5b shows the low temperature (15 K) PL characteristics of the bulk GaSb, GaSb nanofibers and GaSb nanofibers coated with Au thin film and annealed at 600°C for 10 min. There was no PL peak observed from the bulk GaSb, but both the annealed nanofibers and the Au-coated nanofibers exhibited two PL peaks (at 400 and 540 nm), which could be attributed to Ga or oxygen-related vacancy defects. Similarly, two peak results were also obtained in Sinha's work on β-Ga_2_O_3_ 3D microstructures [29]. The nanofiber sample coated with Au (Figure 5b-2) possesed a higher PL intensity than that of the plain annealed nanofibers (Figure 5b-1), which is probably due to surface plasmon effects [30].

## Conclusions

In summary, focused Ga^+^ ion, broad Kr^+^ ion and broad Au^+^ ion beam irradiation were used to fabricate nanofibers on the surface of bulk GaSb. Raman scattering shows that the LO phonon mode of GaSb decreases after ion beam irradiation. A new mode is observed around ~155 cm^-1^ both from unannealed and annealed nanofiber samples. The mode is related to the A_1g_ mode of Sb–Sb bond vibration. Room temperature PL characteristics present an enhancement from the annealed GaSb nanofiber samples compared with the bulk. Quantum confinement effects are discussed in regard to the blue shift of the bandgap. Low temperature (15 K) PL characteristics of the annealed nanofibers show a blue emission peaking at 420 nm and green emission peaking at 550 nm, which may be attributed to atomic defects in the nanostructures, such as oxygen vacancies, gallium vacancies and gallium–oxygen vacancy pairs. Higher PL intensities were obtained from the annealed GaSb fibers coated with an Au thin film, which may be due to surface plasmon effects. The enhanced PL and new band characteristics in the annealed GaSb nanostructures suggest that the irradiation-induced nanofibers may well have vast applications in optoelectronic devices for their unique optical properties.
